# The Role of MRI Lesions in Identifying Secondary Progressive Multiple Sclerosis: A Comprehensive Review

**DOI:** 10.3390/jcm14124114

**Published:** 2025-06-10

**Authors:** Robert-Constantin Anicăi, Alin Ciubotaru, Cristina Grosu, Daniel Alexa, Roxana Covali, Ioana Păvăleanu, Andrei Ionuț Cucu, Amelian Mădălin Bobu, Cristina Mihaela Ghiciuc, Maria Magdalena Leon, Alexandra Maștaleru, Emilian Bogdan Ignat

**Affiliations:** 1 Faculty of Medicine, “Grigore T. Popa” University of Medicine and Pharmacy, 700115 Iași, Romania; anicairobert98@gmail.com; 2 Department of Neurology, “Grigore T. Popa” University of Medicine and Pharmacy, 700115 Iași, Romania; alinciubotaru94@yahoo.com (A.C.); cristina.grosu@umfiasi.ro (C.G.); alexadaniel2004@yahoo.com (D.A.); emilian.ignat@umfiasi.ro (E.B.I.); 3 Clinical Rehabilitation Hospital, 700661 Iași, Romania; maria.leon@umfiasi.ro (M.M.L.); alexandra.mastaleru@umfiasi.ro (A.M.); 4 Department of Radiology, Biomedical Engineering Faculty, “Grigore T. Popa” University of Medicine and Pharmacy, 700115 Iași, Romania; 5 Mother and Child Department, “Grigore T. Popa” University of Medicine and Pharmacy, 700115 Iași, Romania; ioana-m-pavaleanu@umfiasi.ro; 6 Faculty of Medicine and Biological Sciences, University Stefan cel Mare of Suceava, 720229 Suceava, Romania; andrei.cucu@usm.ro; 7 Department of Neurosurgery I, “Prof. Dr. N. Oblu” Clinical Emergency Hospital, Ateneului 2, 700309 Iași, Romania; 8 Department of Cardiology, Sf. Spiridon Hospital, 700111 Iași, Romania; amelian.bobu@gmail.com; 9 Department of Morpho-Functional Sciences II—Pharmacology and Clinical Pharmacology, “Grigore T. Popa” University of Medicine and Pharmacy, 700115 Iași, Romania; cristina.ghiciuc@umfiasi.ro; 10 “Saint Mary” Emergency Children Hospital, 700887 Iași, Romania; 11 Department of Medical Specialties I, “Grigore T. Popa” University of Medicine and Pharmacy, 700115 Iași, Romania

**Keywords:** secondary progressive multiple sclerosis, MRI lesions, gray matter atrophy, slowly expanding lesions, iron rim lesions, spinal cord lesions

## Abstract

Secondary Progressive Multiple Sclerosis (SPMS) represents a challenging phase of multiple sclerosis, marked by gradual neurological decline and reduced inflammatory activity. In recent years, magnetic resonance imaging (MRI) has become essential for characterizing the neurodegenerative changes underlying SPMS, including white and gray matter damage, brain atrophy, slowly expanding lesions, and iron rim lesions. This narrative review aims to synthesize the current knowledge on established and emerging MRI biomarkers relevant to SPMS, with a particular focus on their diagnostic, prognostic, and therapeutic implications. This review discusses key themes, such as the shift from inflammatory to neurodegenerative mechanisms, the role of advanced imaging techniques, and the limitations of conventional MRI in detecting smoldering disease. In doing so, it identifies current gaps in evidence, including the need for standardized imaging protocols and large-scale longitudinal studies. A clearer understanding and application of MRI biomarkers may facilitate earlier diagnosis, more tailored treatment strategies, and improved outcomes in patients with SPMS.

## 1. Introduction

### 1.1. General Context

Secondary Progressive Multiple Sclerosis (SPMS) is a form of multiple sclerosis (MS) characterized by the progressive accumulation of neurological disability following an initial relapsing-remitting phase. According to Ciubotaru et al. [1], SPMS is defined by the transition from relapsing-remitting multiple sclerosis (RRMS) to a phase in which neurological decline continues independently of relapses. This progression is associated with increasing motor and cognitive deficits, which significantly impact the patient’s quality of life [2,3]. Multiple sclerosis (MS) is a chronic immune-mediated neurological disorder that follows distinct clinical courses. The most common initial presentation is RRMS, which is characterized by episodic neurological dysfunction followed by partial or complete recovery [4]. Over time, approximately 50–70% of untreated RRMS patients transition to SPMS within 10–20 years of disease onset, a phase marked by gradual and often irreversible neurological deterioration in the absence of new inflammatory relapses [5,6,7].

Understanding the epidemiological trends and risk factors associated with this transition is essential for improving prognostication and optimizing therapeutic strategies. These factors include age at onset, sex, early disability accumulation, and MRI biomarkers, such as lesion burden and brain atrophy [3,6,8]. Additionally, SPMS is linked to substantial declines in health-related quality of life due to cumulative physical, cognitive, and psychosocial burdens, underscoring the urgent need for early identification and targeted interventions [2,9].

### 1.2. Incidence and Risk of Transition from RRMS to SPMS

The transition from RRMS to SPMS occurs at highly variable rates among individuals. Large-scale epidemiological studies indicate that approximately 50% of RRMS patients convert to SPMS within 19–20 years of disease onset, with the risk increasing with disease duration [2]. However, more recent data suggest that this proportion may be decreasing due to earlier diagnosis and the widespread use of disease-modifying therapies (DMTs) [10]. The introduction of high-efficacy DMTs has indeed been linked to a significantly reduced risk of conversion to SPMS in treated populations [11]. In addition to treatment, early disease factors, such as a heavy initial MRI lesion burden, have been associated with earlier SPMS onset, while certain modifiable environmental influences (e.g., regional differences in MS prevalence) may also affect progression rates [12,13].

Several factors influence the rate of transition from RRMS to SPMS. Studies have shown that the risk of progression is higher in individuals with a high relapse rate during the RRMS phase, particularly in the first five years after diagnosis. Moreover, the transition was more frequent in patients with older age at RRMS onset, with those diagnosed after the age of 40 years experiencing a more rapid conversion to SPMS [3]. Another key finding from epidemiological data is that the female sex, although associated with a higher incidence of RRMS, does not confer a protective effect against progression to SPMS [2].

### 1.3. Aim of the Review

The objective of this review was to comprehensively explore the utility of MRI-derived biomarkers in the diagnosis and prognosis of SPMS. We aimed to critically analyze the current research on MRI-detected lesions, their characteristics, and their evolution over time in SPMS [4]. We also aimed to evaluate the role of brain atrophy, both global and regional, and its correlation with clinical measures of disability [14]. Furthermore, we examine the potential of advanced imaging techniques, such as diffusion tensor imaging (DTI) and magnetization transfer imaging (MTI), in detecting subtle tissue changes in SPMS [15]. A key focus will be on evaluating the strength of the evidence for each biomarker, considering factors such as sensitivity, specificity, and predictive values. Finally, we aim to analyze how these MRI biomarkers can improve the early diagnosis of SPMS, guide treatment decisions, and serve as surrogate endpoints in clinical trials.

## 2. Materials and Methods

As this manuscript adopts a narrative review framework, we did not perform a systematic literature search. Instead, we conducted targeted queries across platforms, including PubMed (U.S. National Library of Medicine. PubMed. Available at: https://pubmed.ncbi.nlm.nih.gov, accessed on 23 April 2025) and Web of Science (Clarivate Analytics. Web of Science. Available at: https://www.webofscience.com, accessed on 23 April 2025), using keywords such as “secondary progressive multiple sclerosis,” “MRI and SPMS”, “MRI lesions”, “cortical lesions”, “brain atrophy”, “spinal cord”, and “iron rim lesions”. To ensure comprehensive coverage, we subsequently performed citation tracking by examining the reference lists of the included articles (backward citation search) and used citation index tools within PubMed, Web of Science, and Scopus to identify more recent studies that cited these key articles (forward citation search). Although this approach has the advantage of highlighting relevant clinical and imaging developments, it also introduces the limitation of potential selection bias inherent to non-systematic review methods.

## 3. Pathophysiology of SPMS

### 3.1. Progression from RRMS to SPMS: Changes in Disease Mechanisms

MS is a chronic demyelinating disease characterized by an interplay between inflammation-driven damage and neurodegeneration. In the early stages, RRMS is dominated by focal inflammatory lesions, whereas SPMS shows a shift towards neurodegenerative mechanisms, with a decline in relapse and an increase in continuous neurological deterioration. The transition from RRMS to SPMS is associated with changes in the underlying pathophysiology, affecting immune response, neuronal integrity, and glial interactions [4,5]. In Table 1, we summarize these changes by distinguishing between inflammatory and neurodegenerative mechanisms.

### 3.2. Inflammatory Mechanisms in RRMS

RRMS is fundamentally an inflammatory autoimmune disease characterized by recurrent demyelinating episodes driven by peripheral immune cell infiltration into the central nervous system (CNS). A critical event in this process is the disruption of the blood–brain barrier (BBB), which facilitates the entry of autoreactive T-cells and B-cells into the CNS parenchyma, where they initiate a cascade of immune-mediated damage [5]. Among these, T-helper 17 (Th17) cells play a central role in producing interleukin-17 (IL-17), a cytokine that amplifies inflammation and facilitates additional immune cell recruitment [5]. B cells contribute to the production of antibodies and the release of pro-inflammatory cytokines that promote lesion development and demyelination [5]. Furthermore, activated macrophages and microglia release tumor necrosis factor-alpha (TNF-α) and interleukin-6 (IL-6), contributing significantly to myelin and axonal damage [4,5]. This immune-driven pathology manifests clinically as relapses, in which acute inflammation leads to transient neurological symptoms that may partially resolve due to remyelination mechanisms. The cumulative effect of these inflammatory insults lays the groundwork for subsequent neurodegenerative processes characteristic of progressive disease phases.

### 3.3. Transition from RRMS to SPMS: A Shift in Mechanisms

Over time, RRMS evolves into SPMS, a phase characterized by a gradual decline in neurological function that is largely independent of overt relapse. This transition reflects a fundamental shift in disease mechanisms: inflammation becomes compartmentalized within the central nervous system (CNS), and neurodegenerative processes begin to dominate [4]. One of the central changes is a reduction in peripheral immune cell infiltration, leading to a decreased frequency of clinical relapses despite ongoing disease activity [4,5].

Meanwhile, chronic microglial activation persists within the CNS, maintaining a low-grade, smoldering inflammatory milieu that promotes sustained tissue damage in the absence of new inflammatory foci [16]. Another key contributor is mitochondrial dysfunction, which results in axonal energy failure, increased oxidative stress, and progressive neuronal loss in both white and gray matter structures [4,16]. Concurrently, the failure of remyelination due to oligodendrocyte precursor cell dysfunction limits the repair capacity and leads to irreversible demyelination and axonal degeneration [16]. Collectively, these mechanisms underpin the progressive motor, sensory, and cognitive decline observed in SPMS, marking a clinical and pathological divergence from the relapsing course of the early disease.

### 3.4. Neurodegenerative Mechanisms in SPMS

In SPMS, neurodegeneration gradually overtakes acute inflammation as the predominant driver of disability accumulation. One of the key pathological features distinguishing SPMS from relapsing-remitting MS (RRMS) is chronic microglial activation. Unlike the transient, relapse-associated inflammation seen in RRMS, microglia in SPMS remain persistently activated, promoting sustained injury to myelin and axons, even in the absence of peripheral immune cell infiltration [16]. Concurrently, axonal loss becomes widespread, largely driven by mitochondrial dysfunction. Energy failure and increased oxidative stress within neurons and axons contribute to progressive neuroaxonal degeneration and apoptosis, further exacerbating neurological decline [4,16]. Cortical atrophy also becomes increasingly prominent in SPMS, particularly affecting the frontal, temporal, and parietal lobes. This widespread cortical thinning correlates with both physical and cognitive impairment and serves as a powerful prognostic indicator of disease progression in patients with PSP. A defining radiological hallmark of SPMS is the emergence of slowly expanding or “smoldering” lesions (SELs) —chronically active plaques characterized by a persistent rim of paramagnetic material, often visible on susceptibility-weighted imaging. These lesions reflect compartmentalized inflammation maintained by iron-laden microglia and macrophages and are strongly associated with cumulative disability and cognitive deterioration over time [4,17,18,19]. While the frequency of new gadolinium-enhancing lesions diminishes in SPMS, smoldering lesions persist or expand, indicating an ongoing pathology beneath a seemingly quiescent surface. Importantly, a subset of SPMS patients—estimated at around one-third—may still show signs of MRI activity, including new or contrast-enhancing lesions, despite the absence of clinical relapses [20]. Nevertheless, even in “non-active” SPMS, disability progresses at a comparable rate to that with inflammatory activity, highlighting that progression in SPMS is often driven by mechanisms independent of acute inflammation [21].

## 4. MRI Lesions and Biomarkers in SPMS: Current Insights into Disease Progression

### 4.1. Current Knowledge Gap: Why SPM Is a Distinct Challenge in MS Research and Clinical Practice

SPMS is characterized by steadily worsening neurological function, often in the absence of distinct relapses [22]. This makes SPMS far more difficult to identify promptly than RRMS, which is typically diagnosed based on the presence of discrete relapses and corresponding MRI findings. In SPMS, subtle and continuous progression of disability can lead to retrospective diagnoses, delaying potentially beneficial therapeutic changes. Even proposed diagnostic frameworks, such as those correlating EDSS scores with relapse frequency [23], do not fully capture the nuanced transition from an inflammatory to a predominantly neurodegenerative disease.

Chronic compartmentalized inflammation in the central nervous system (CNS) is fundamental to SPMS [24]. Meningeal B-cell follicles, ongoing microglial activation, and lower levels of overt blood–brain barrier disruption result in a ‘smoldering’ disease course that conventional MRI techniques often fail to detect reliably. Consequently, many patients endure suboptimal therapy throughout this insidious shift from RRMS to SPMS, underscoring the urgent need for better-defined MRI biomarkers that can detect these chronic neurodegenerative changes. Advanced imaging modalities that recognize SELs or iron-laden rims may help bridge this gap, but systematic validation in diverse cohorts is still lacking [25]. This gap in evidence-based imaging strategies leaves patients with SPMS at risk of advanced disability before current clinical tools signal the need for therapeutic escalation.

### 4.2. Factors Contributing to SPMS Progression

The progression from RRMS to SPMS is influenced by various clinical, radiological, genetic, environmental, and molecular factors. Understanding these contributors is critical for identifying patients at high risk and tailoring early therapeutic strategies.

One of the most robust clinical predictors of disease progression is the rate of early disability accumulation. Patients who reach an Expanded Disability Status Scale (EDSS) score of 3.0 within five years of onset or 4.0 within ten years have a significantly higher likelihood of transitioning to SPMS earlier than those with a more indolent disease course [3]. Rapid disability progression in the early years of RRMS suggests a more aggressive inflammatory burden and limited compensatory mechanisms [3]. MRI biomarkers also provide critical prognostic information about the disease. A high lesion burden on T2-weighted imaging, early presence of T1-hypointense lesions (“black holes”), and pronounced atrophy, especially of the thalamus and cortical gray matter, are all associated with faster progression to SPMS [2,6,8]. The presence of spinal cord lesions further increases the likelihood of progression due to their direct impact on the motor pathways [6]. Additionally, a high frequency of gadolinium-enhancing lesions in early RRMS correlates with greater inflammatory activity and is associated with a more rapid transition to SPMS [2].

Sex and age at disease onset are important demographic factors. Although RRMS is more prevalent in females, the male sex is linked to a more aggressive disease course and earlier conversion to SPMS, possibly due to reduced neuroprotective hormonal effects [2]. Similarly, older age at disease onset is associated with a shorter time to SPMS, likely due to diminished remyelination capacity and neuroplasticity in aging neural tissue [2].

Lifestyle and environmental factors also modulate the disease progression. Among these, smoking is one of the most consistently reported risk factors. Smokers with MS progress to SPMS approximately five years earlier than non-smokers, and the effect is dose-dependent. Smoking is thought to exacerbate disease progression through mechanisms such as increased oxidative stress, promotion of chronic inflammation, and impaired blood–brain barrier repair [2]. Comorbidities, especially vascular risk factors such as hypertension, diabetes, and hyperlipidemia, have also been shown to accelerate disability accumulation and cognitive decline in patients with MS. These conditions may potentiate microvascular ischemia and amplify neuroinflammatory cascades, worsening the underlying CNS pathology [6,7].

From a genetic perspective, carriers of the HLA-DRB1*15:01 allele not only face a higher risk of developing MS but may also experience a more severe disease phenotype and a faster transition to SPMS [3]. Although this association is well documented, ongoing studies are investigating the roles of additional loci and polygenic risk scores in predicting disease progression.

Emerging research has identified potential molecular and immunological markers of its progression. Elevated serum neurofilament light chain (NfL) levels have been associated with axonal damage and correlated with future disability in both RRMS and SPMS [17]. Moreover, alterations in vitamin D metabolism, often marked by deficiency, have been implicated in increased disease activity and accelerated progression, although the findings remain heterogeneous [26]. Additionally, the gut microbiome and prior Epstein–Barr virus (EBV) infection have garnered attention as potential modulators of disease progression. While EBV is universally present in patients with MS and may trigger early autoimmunity, its direct role in SPMS progression remains poorly defined [26]. Similarly, dysbiosis of the gut microbiota may influence neuroinflammation; however, further mechanistic studies are needed to elucidate the causal links [26].

### 4.3. Motor Progression in SPMS

Motor progression in SPMS is characterized by a gradual decline in mobility and muscle function, primarily due to ongoing neurodegeneration and cumulative demyelination. Studies have indicated that patients with SPMS experience a steady increase in disability, with walking impairment being one of the earliest and most prominent features [3]. Declines in ambulation are strongly correlated with the progression of spinal cord atrophy and axonal loss, which contribute to weakness, spasticity, and impaired coordination [2].

One of the key epidemiological findings is that early disability accumulation in RRMS is a strong predictor of motor progression in SPMS. Patients who reach an EDSS score of 4.0 (requiring assistance for prolonged walking) within the first ten years of disease onset have a significantly higher risk of rapid motor decline in SPMS [3]. Additionally, studies have shown that a greater lesion load in the spinal cord on MRI is associated with faster progression of gait impairment and an increased risk of wheelchair dependence within 10–15 years of SPMS onset [2].

Factors such as age at disease onset and sex also influence motor progression. An older age at RRMS diagnosis has been linked to a shorter time to SPMS conversion and a faster accumulation of motor disability [2]. Additionally, male patients tend to experience more severe motor impairment than female patients, likely due to differences in neuroprotective mechanisms and hormonal influences [3].

### 4.4. Cognitive Decline in SPMS

Cognitive impairment is a major aspect of SPMS progression, affecting various domains, including memory, executive function, and information-processing speed. Epidemiological data suggest that up to 70% of patients with SPMS experience significant cognitive decline, which worsens over time due to cortical and subcortical atrophy [2]. Unlike RRMS, where cognitive dysfunction is often episodic and linked to relapses, in SPMS, cognitive dysfunction progresses steadily, reflecting ongoing neurodegeneration rather than inflammation [3].

MRI studies have shown that brain atrophy, particularly in the frontal and temporal lobes, is a key factor in cognitive decline. Gray matter atrophy is strongly correlated with worsening memory and reduced processing speed, while subcortical atrophy, including the thalamus, is associated with executive dysfunction and attention deficits [2]. Furthermore, the presence of chronic T1 hypointense lesions (“black holes”) has been linked to irreversible axonal damage and worsening of cognitive function [3].

Several risk factors contribute to the accelerated cognitive decline in SPMS. Older age at SPMS onset is associated with more severe cognitive impairment, while a higher lesion load in early RRMS predicts worse cognitive outcomes in the progressive phase [2]. Additionally, smoking and comorbid vascular diseases, such as hypertension and diabetes, have been shown to exacerbate cognitive dysfunction by increasing neuroinflammation and vascular damage [3].

The SPMS phase represents an advanced stage of multiple sclerosis, characterized by the progressive accumulation of neurological disabilities without distinct relapses. The impact of this phase on the quality of life of patients is significant and multidimensional. Deterioration of physical function is a defining feature of SPMS and has a direct impact on the quality of life. Reduced mobility, coordination difficulties, and balance problems limit daily activities, reducing independence and social participation. Cognitive dysfunction, including problems with memory, concentration, and information processing, is common in SPMS. This affects the ability to work, maintain social relationships, and carry out recreational activities, having a negative impact on the quality of life [1,27].

Fatigue is a common and debilitating symptom of SPMS that is often underestimated. The persistent feeling of physical and mental exhaustion limits the ability to carry out daily and social activities, significantly affecting the quality of life. Chronic pain is another frequent problem in SPMS, with a negative impact on the quality of life. The pain can be neuropathic, muscular, or articular and can affect mobility, sleep, and emotional state [27].

Depression and anxiety are more common in patients with SPMS than in the general population. Coping with progressive disability, chronic pain, and fatigue can contribute to the appearance of mental health problems, affecting the quality of life. The social impact of SPMS is significant, including difficulties in maintaining a job, loss of financial independence, and social isolation. These problems can affect self-esteem, interpersonal relationships, and quality of life [27].

Quality of life is influenced by all the factors mentioned above. Patients with SPMS often report a lower quality of life than those with other forms of MS or the general population.

### 4.5. Utility of MRI in Diagnosis and Monitoring

1. Brain Atrophy Measurements

Global and regional brain volume loss is a key marker of SPMS conversion, as it strongly correlates with accumulating disability. Progressive atrophy of deep gray matter structures, particularly the thalamus, is predictive of disease worsening. Recent large cohort analyses have confirmed that early deep gray matter atrophy (notably thalamic atrophy) is a major driver of long-term disability progression and conversion to SPMS [8].

2. Cortical Lesions and Thinning

Unlike RRMS, in which white matter lesions are dominant, SPMS is characterized by more extensive cortical demyelination. Advanced imaging techniques have highlighted the importance of cortical lesion burden and leptomeningeal contrast enhancement as markers of SPMS conversion. In progressive MS, the presence of leptomeningeal enhancement on MRI—an in vivo marker of meningeal inflammation—has been associated with accelerated cortical gray matter atrophy over time [28].

3. Atrophied Lesion Volume (ALV)

A newly recognized biomarker, ALV, quantifies the transition of demyelinated lesions into cerebrospinal fluid, representing irreversible neurodegeneration. It has shown stronger correlations with clinical disability than traditional lesion counts.

4. Slowly Expanding Lesions (SELs)

These smoldering lesions, which display a paramagnetic rim on susceptibility-weighted imaging, indicate chronic active inflammation and are more prevalent in patients with SPMS. SELs are associated with greater tissue destruction and long-term disability progression.

5. Spinal Cord Involvement

While spinal cord atrophy is difficult to measure with standard MRI, emerging imaging protocols now allow for better assessment of spinal cord damage, which is a strong predictor of physical disability in SPMS [20]. Indeed, pronounced spinal cord atrophy on MRI can precede the clinical onset of SPMS, serving as an early indicator of a progressive disease [29].

6. Visualization of Axonal Loss

**Figure 1:**
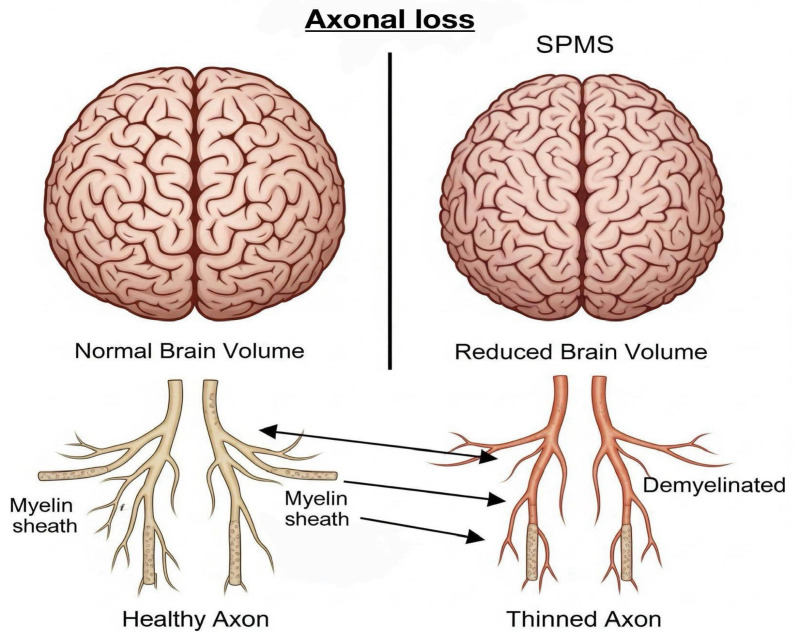
Axonal loss due to demyelination. Over time, this leads to brain atrophy and irreversible neurological deficits.

Axonal loss, a critical driver of disability progression in SPMS, is associated with irreversible neurological deficits in patients with SPMS. MRI indirectly detects axonal loss by evaluating brain atrophy, lesion burden, and altered diffusion patterns. Brain atrophy, reflected by reductions in brain volume, particularly in the grey matter (GM) and white matter (WM), is a hallmark of significant axonal degeneration and neuronal loss. Quantitative MRI techniques, including the normalized brain volume (NBV), normalized cortical GM volume (NCGMV), and normalized WM volume (NWMV), are used to assess this loss. It has been shown that patients with SPMS exhibit more pronounced brain volume reduction compared to those with RRMS [6]. Furthermore, diffusion tensor imaging (DTI) offers additional insights into axonal damage through parameters such as fractional anisotropy (FA) and mean diffusivity (MD). These metrics reflect the microstructural integrity within the white matter tracts. In SPMS, decreased FA values in regions such as the fornix and medial lemniscus have been correlated with cognitive impairment and disease progression, underscoring the clinical relevance of axonal integrity as revealed by advanced imaging techniques, Figure 1 [7].

7. Visualization of Demyelination

Demyelination, the hallmark pathological feature of multiple sclerosis (MS), can be identified using both conventional MRI sequences and specialized imaging techniques. T2-weighted MRI sequences typically reveal hyperintense lesions that correspond to regions of demyelination, edema, and inflammation. While an increased lesion load is a common finding in RRMS, in SPMS, the lesions often become more diffuse and slowly expand rather than appear as new, discrete foci [30]. In contrast, T1-weighted sequences may show hypointense lesions, known as “black holes”, which reflect areas of severe and often irreversible demyelination and axonal loss. These T1-hypointense lesions are strongly associated with clinical disability and cognitive impairment in patients with SPMS. Their volume and persistence over time are indicative of poor prognosis [26].

8. Visualization of Chronic Lesions

Chronic lesions, especially SELs, are predominant features of SPMS and reflect the presence of ongoing low-grade inflammation and neurodegeneration. SELs are visualized on MRI as lesions with persistent, gradually expanding borders that evolve over the years, indicating continuous demyelination and axonal injury. These lesions are strongly associated with clinical progression, often independent of new inflammatory activity [30]. Leptomeningeal enhancement (LME), which can be detected using post-contrast fluid-attenuated inversion recovery (FLAIR) sequences, serves as a marker of chronic meningeal inflammation. The presence of LME correlates with cortical demyelination and neurodegeneration, particularly in progressive forms of MS [31].

### 4.6. Importance of Early MRI Detection for Patient Outcomes

Early detection of multiple sclerosis (MS) progression from the RRMS to SPMS using MRI plays a critical role in improving clinical outcomes and patients’ quality of life. Timely recognition of subtle pathological changes using advanced MRI techniques can significantly influence disease management, therapeutic decision-making, and prognosis, potentially reducing the burden of long-term disability.

1. Identification of Patients at Risk for Early Progression.

MRI allows clinicians to identify patients at a higher risk of rapid progression even before the pronounced clinical manifestations. Specific MRI features, such as increased brain atrophy rates, higher lesion burden, and the presence of SELs, are critical indicators for predicting early progression to SPMS [6,30]. Notably, Koch et al. found that brain volume loss, especially involving gray matter, strongly correlates with physical disability and cognitive impairment, emphasizing its value as an early prognostic indicator [6].

2. Improved Therapeutic Strategies and Timely Interventions.

Early MRI detection facilitates timely therapeutic interventions, which are crucial for modifying disease progression. Identifying patients with progressive neurodegeneration allows clinicians to proactively adjust treatments, potentially delaying or preventing severe disability. For instance, the early identification of subpial demyelination or leptomeningeal inflammation, detectable through advanced MRI sequences, may guide more aggressive therapeutic strategies, including the early initiation of disease-modifying therapies (DMTs) or neuroprotective agents [30,31].

3. Prevention or Delay of Cognitive Decline.

Early identification of MRI markers for cognitive impairment significantly enhances patient management. Progressive MS is frequently associated with substantial cognitive deficits that profoundly affect the quality of life, employment status, and social functioning. Advanced MRI techniques, such as diffusion tensor imaging (DTI), allow the detection of microstructural alterations in critical white matter tracts linked to cognition (e.g., the corpus callosum and fornix). As Mistri et al. emphasized, abnormalities in fractional anisotropy (FA) within these structures strongly correlate with cognitive impairment. Detecting these MRI biomarkers early enables tailored cognitive rehabilitation and targeted pharmacological interventions, enhancing cognitive outcomes and preserving the quality of life [7].

4. Enhanced Monitoring of Disease Activity and Treatment Efficacy.

MRI plays a crucial role in the objective monitoring of disease activity and evaluation of treatment efficacy. Through regular MRI assessments, clinicians can detect subclinical disease activity and inform therapeutic decisions. Filippi et al. highlighted the importance of imaging biomarkers, like new T2 lesions or gadolinium-enhancing lesions, as indicators of ongoing inflammatory activity. Furthermore, monitoring atrophy rates provides insight into neurodegenerative progression, allowing clinicians to evaluate the effectiveness of neuroprotective therapies and adjust treatments accordingly [30].

5. Prognostication and Patient Counseling.

Early detection of progressive MRI features enables accurate prognostication, facilitating informed discussions between clinicians and patients regarding the expected disease trajectory and outcomes. A clear MRI-based understanding of disease status empowers patients by setting realistic expectations, guiding lifestyle adaptations, and optimizing psychological preparedness for potential disease progression. Koch et al. found correlations between MRI features, including lesion burden and brain atrophy, and significant clinical outcomes, underscoring their importance in patient counseling and management [6].

6. Economic and Social Implications.

Early and accurate detection using MRI can significantly impact healthcare resource utilization and societal costs associated with MS. By delaying the progression of disability, patients maintain higher functional independence, reducing direct healthcare costs (e.g., hospitalization and supportive care) and indirect costs (e.g., loss of productivity and caregiver burden). Thus, early identification of at-risk patients through MRI-based assessments yields substantial economic benefits alongside improved patient outcomes [30].

### 4.7. Imaging Biomarkers in SPMS

MRI has become an indispensable tool for the diagnosis, monitoring, and prognostication of multiple sclerosis (MS). In the context of SPMS, where neurodegeneration gradually overtakes inflammatory activity, the role of MRI extends far beyond the detection of active lesions. Instead, it facilitates the visualization of chronic pathological processes—many of which may not be clinically apparent until substantial disability has accumulated. As such, MRI biomarkers are increasingly used to characterize the underlying tissue damage that drives irreversible neurological decline in patients with SPMS [4,5,6]. Several classes of MRI biomarkers are now recognized for their relevance to SPMS. Structural MRI remains foundational, with T2-weighted imaging used to quantify the total lesion burden and T1-weighted imaging revealing hypointense “black holes” that correspond to areas of severe axonal loss. These traditional measures are useful for tracking the long-term disease burden, although they lack specificity for progressive pathology. In SPMS, progressive brain atrophy—particularly of the cortical gray matter and deep gray matter nuclei such as the thalamus—has emerged as a highly predictive marker of sustained disability. Cortical thinning and regional atrophy strongly correlate with both physical impairment and cognitive decline, often preceding visible clinical deterioration [6,9]. Recently, advanced imaging biomarkers have become the focus of SPMS research and clinical practice. SELs, which represent chronically active white matter plaques with ongoing inflammation at their margins, are now considered the hallmarks of smoldering disease activity. These lesions typically lack gadolinium enhancement but show peripheral expansion over time on follow-up imaging, reflecting the persistent microglial activation and progressive demyelination. Their presence is significantly associated with future disability and brain volume loss [4,17]. Similarly, iron rim lesions—characterized by paramagnetic borders visible on susceptibility-weighted imaging (SWI) or quantitative susceptibility mapping (QSM)—are indicative of iron-laden activated microglia and macrophages. These lesions offer a promising window into compartmentalized inflammation and correlate with aggressive disease progression [18,32]. Quantitative approaches have also refined the characterization of lesions. The concept of atrophied lesion volume (ALV), which refers to the transformation of demyelinated lesions into cerebrospinal fluid-filled cavities, offers a biomarker for irreversible tissue destruction. ALV has shown stronger correlations with clinical disability than lesion counts alone and provides a clearer index of cumulative neurodegeneration [8]. In addition to structural imaging, several microstructural and metabolic MRI techniques have been integrated into SPMS evaluation. Diffusion tensor imaging (DTI) assesses the integrity of white matter tracts by measuring the diffusion of water molecules. Reductions in fractional anisotropy (FA) and elevations in mean diffusivity (MD) are indicative of axonal loss and have been linked to both motor and cognitive deficits [7,33]. Magnetization transfer imaging (MTI) quantifies the interaction between macromolecules and water protons, thereby reflecting myelin density. Decreased magnetization transfer ratios (MTR) have been observed in both lesions and normal-appearing brain tissue in SPMS, suggesting a more diffuse pathology than that visible on standard scans [18,34]. Magnetic resonance spectroscopy (MRS) provides metabolic insights and reveals changes in brain biochemistry that parallel clinical decline. For example, reduced levels of N-acetyl aspartate (NAA), a marker of neuronal integrity, and elevated choline, associated with membrane turnover, have been observed in patients with SPMS and correlate with disease severity (Table 2) [34].

### 4.8. MRI Lesion Types in SPMS

1. White Matter Lesions.

In SPMS, MRI is an essential tool for characterizing disease progression by identifying and monitoring white matter lesions. These lesions typically manifest as hyperintense areas on T2-weighted MRI scans, representing areas of demyelination, axonal loss, and gliosis. T2 hyperintense lesions are a hallmark of multiple sclerosis and serve as critical biomarkers for evaluating disease burden and progression.

White matter lesions in SPMS are characterized by their initial presentation as active lesions that may be enhanced with gadolinium contrast on T1-weighted imaging, indicating blood-brain barrier disruption and acute inflammatory activity. However, over time, these active lesions typically transition into chronic demyelinated plaques, identifiable as non-enhancing stable lesions that persistently appear hyperintense on T2-weighted MRI and hypointense on T1-weighted MRI [35].

Chronic white matter lesions in SPMS can become (SELs), which represent a significant proportion of the total lesion volume and are specifically associated with progressive neurological disability. SELs exhibit gradual expansion without evidence of acute inflammatory activity, such as gadolinium enhancement, and their volume is strongly correlated with clinical deterioration, as measured by the Expanded Disability Status Scale (EDSS) and other functional assessments [35].

Histopathologically, chronic lesions often display ongoing neurodegeneration and axonal loss despite the absence of marked inflammation. This suggests that the pathology underlying these chronic lesions includes mechanisms independent of acute inflammatory processes, potentially involving innate immune system activation, neurodegenerative processes, and persistent tissue damage mediated by activated microglia and macrophages [36].

Moreover, the accumulation of white matter lesion volume has been consistently correlated with increasing levels of physical disability in patients with SPMS. Higher lesion loads are indicative of more severe neurological deficits and predict worse clinical outcomes [37].

**Figure 2:**
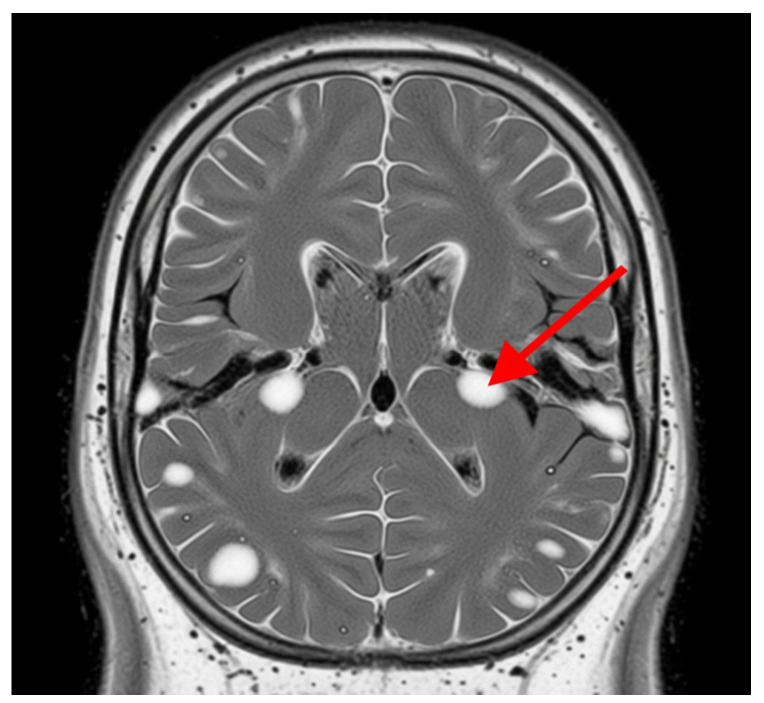
White matter lesions. The grey area represents the brain tissue, and the bright white areas represent the white matter lesions. These lesions are typically hyperintense (brighter) on T2-weighted MRI scans and represent areas of damage to the myelin sheath, which insulates nerve fibers in white matter. The red arrow indicates one of these distinct bright white lesions. In SPMS, these lesions indicate demyelination, axonal loss, and gliosis.

Advanced MRI techniques have enhanced the capacity to identify and characterize these lesions, providing valuable insights into their evolution and their relationship with clinical outcomes. The persistent and expanding nature of white matter lesions highlights their central role in SPMS pathophysiology and underscores the importance of longitudinal MRI studies for monitoring disease progression and therapeutic response, Figure 2 [38].

2. Gray Matter Lesions.

Gray matter (GM) lesions, particularly in the cortical and subcortical regions, significantly contribute to the pathology of SPMS, presenting distinctive challenges for detection and clinical interpretation.

Importance of cortical and subcortical gray matter involvement.

Gray matter atrophy, particularly affecting the cortical gray matter (cGM) and deep gray matter structures like the thalamus, is strongly linked to disability accumulation and cognitive decline in patients with SPMS. Studies such as Arnold et al. have demonstrated that cortical gray matter and thalamic volume loss correlate significantly with the progression of neurodegeneration, independent of inflammatory lesions typical of relapsing forms of the disease. These changes reflect the ongoing neurodegenerative processes that dominate the pathology in the progressive stages of MS beyond the inflammation-driven damage observed in relapsing MS [9].

Involvement of the cortical regions and thalamus not only correlates with physical disability but also cognitive impairment, highlighting their prognostic significance in SPMS. Specifically, cortical atrophy is associated with a progressive decline in cognitive functions, such as processing speed and executive functions [39]. Furthermore, resting-state functional connectivity studies have emphasized the role of gray matter changes in altering brain network dynamics, further contributing to cognitive and motor impairments [17].

Gray matter lesions present unique challenges due to their subtle appearance on conventional MRI techniques. Traditional T2-weighted MRI, which is effective in detecting white matter lesions, is less sensitive to gray matter pathology, thus underestimating its true extent. Advanced imaging techniques, such as Magnetization Transfer Ratio (MTR), Double Inversion Recovery (DIR), and quantitative susceptibility mapping (QSM), enhance the detection and quantification of gray matter lesions. The MTR technique, for instance, has been instrumental in revealing myelin integrity changes in normal-appearing brain tissues (NABT), including cGM and normal-appearing white matter (NAWM), which are not visible on standard MRI scans [9].

Additionally, susceptibility-based imaging methods have been increasingly used to detect cortical lesions characterized by chronic iron-containing inflammatory rims, which are significant markers of ongoing inflammation and neurodegeneration [32]. Despite these advancements, the accurate detection and consistent quantification of gray matter lesions in clinical practice remain challenging due to the subtlety of pathological changes, technical variability between MRI scanners, and the need for specialized imaging protocols that are not universally available or standardized across clinical settings [18].

Thus, while cortical and subcortical gray matter involvement has emerged as a critical aspect of SPMS pathology, necessitating sensitive imaging modalities for accurate detection, considerable efforts are still required to standardize these advanced imaging techniques for routine clinical applications.

3. Iron Deposition.

Iron deposition, particularly in deep gray matter structures, is increasingly recognized as an important pathological feature of SPMS, reflecting chronic inflammation and neurodegeneration. Advanced MRI techniques, such as Susceptibility Weighted Imaging (SWI) and Quantitative Susceptibility Mapping (QSM), have significantly enhanced the visualization and understanding of iron accumulation in these brain regions.

Iron accumulation is commonly observed within the basal ganglia, thalamus, and at the rims of chronic active lesions and correlates strongly with disease severity and progression in SPMS [32]. SWI effectively detects paramagnetic substances like iron, highlighting lesions with iron-containing inflammatory rims indicative of chronic microglial activation and ongoing tissue injury. QSM further refines this by providing quantitative measures of iron levels, facilitating precise monitoring of disease progression, and potentially predicting disability outcomes [17].

These advanced imaging modalities are particularly useful in distinguishing chronic active lesions—characterized by persistent inflammation and iron-laden macrophages—from inactive lesions, thereby contributing significantly to prognosis and treatment strategies [18]. Hence, incorporating iron-sensitive MRI techniques into routine clinical practice holds promise for more accurate assessment and management of SPMS.

4. Chronic Active Lesions (“Smoldering Lesions”).

Chronic active lesions, commonly termed “smoldering lesions”, are critical pathological hallmarks of SPMS. These lesions are characterized by a persistent inflammatory rim around their edges, which is distinctively visible on SWI. The appearance of these hypointense rims on SWI is primarily due to iron accumulation within activated microglia and macrophages, signifying ongoing inflammatory activity despite the absence of typical acute inflammatory episodes [32].

The identification of rim lesions through advanced MRI techniques, particularly SWI, has significant clinical implications, as these lesions are strongly correlated with ongoing neurodegeneration and clinical progression. Studies have demonstrated that patients with a higher burden of chronic active lesions exhibit more pronounced disability accumulation and cognitive deterioration over time [17,18]. Absinta et al. further showed that lesions with paramagnetic rims (indicative of chronic active inflammation) tend to persist or even expand over the years, whereas rimless lesions gradually shrink—reinforcing the link between rimmed lesions and more aggressive disease progression [19].

**Figure 3:**
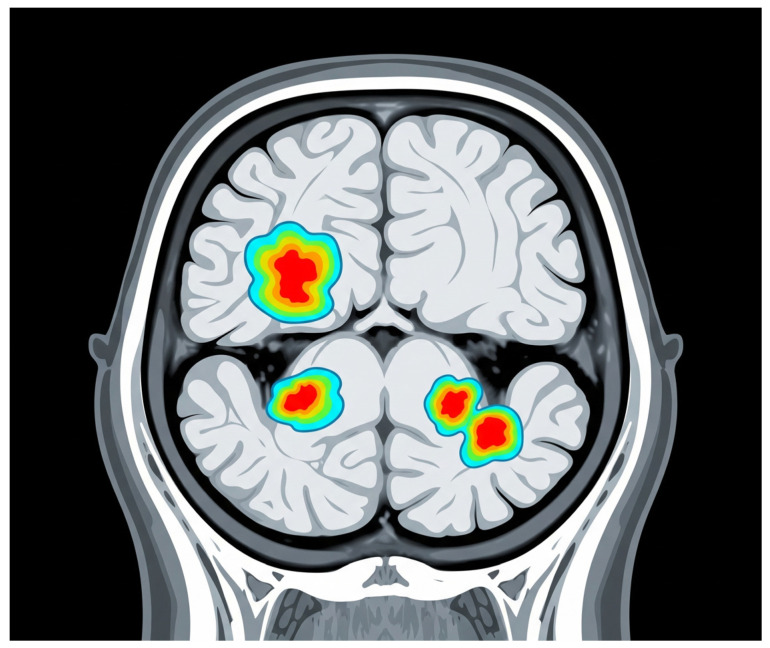
Chronic active lesions. The background grey represents the brain tissue. The areas highlighted with a color gradient (from blue to red) represent chronic active lesions, also known as “smoldering lesions.” These lesions are characterized by persistent, low-level inflammation. Although they may not appear as intensely bright as typical acute white matter lesions, these areas indicate ongoing damage. In more advanced MRI techniques (not directly shown in this illustrative style), these lesions often show a dark rim, indicative of chronic inflammation.

Consequently, smoldering lesions are gaining recognition not only as robust biomarkers for disease progression but also as potential targets for therapeutic intervention. Monitoring their presence and evolution through routine imaging could facilitate timely clinical decision-making and enhance prognostic precision, ultimately contributing to improved patient management in SPMS patients. Overall, these findings support the view that progressive MS is driven by smoldering pathology, prompting proposals for a new framework of MS progression based on underlying mechanisms rather than relapses alone, Figure 3 [40].

5. Spinal Cord Lesions

Spinal cord lesions are frequent and clinically significant in SPMS, manifesting distinct patterns of involvement compared with other MS phenotypes. These lesions often correlate with substantial clinical impairment due to their critical anatomical localization. Spinal cord involvement, especially when extensive, frequently correlates with severe neurological deficits, including motor weakness, sensory disturbances, and autonomic dysfunctions [18].

Clinically, the frequency and extent of spinal cord lesions tend to increase with disease progression and are notably more prevalent and severe in SPMS than in RRMS. This increased burden contributes significantly to disease progression and disability accumulation, reflecting the chronic neurodegenerative aspect characteristic of the secondary-progressive stage [9].

The pathological hallmarks of spinal cord lesions in SPMS include a higher prevalence of diffuse demyelination, axonal loss, and gliosis, differentiating them from lesions predominantly seen in RRMS, which typically exhibit more pronounced inflammatory features and less extensive axonal degeneration [33]. Moreover, the spinal cord lesions observed in SPMS frequently show persistent signal abnormalities on MRI without acute gadolinium enhancement, indicating chronic degenerative rather than active inflammatory processes [17].

### 4.9. Advanced MRI Techniques in SPMS

1. Quantitative MRI Techniques.

Quantitative MRI techniques, including MTI, DTI, and Magnetic Resonance Spectroscopy (MRS), are pivotal in assessing microstructural damage in SPMS. These methods provide insights beyond those of conventional MRI, enabling detailed evaluations of disease progression.

MTI utilizes the exchange of magnetization between protons bound to macromolecules (such as myelin proteins and lipids) and those in the surrounding tissue water. The Magnetization Transfer Ratio (MTR) derived from MTI sequences specifically reflects myelin integrity, although it also correlates with axonal density. Decreased MTR values indicate demyelination and axonal loss, which are prominent in patients with SPMS [36]. Furthermore, reduced MTR values in normal-appearing white and gray matter (NAWM, NAGM) are more pronounced in SPMS than in relapsing forms, highlighting the advanced microstructural pathology characteristic of SPMS [33].

DTI measures the diffusion of water molecules in tissues and provides fractional anisotropy (FA) and mean diffusivity (MD) metrics, both of which are indicators of microstructural integrity. DTI studies have demonstrated increased MD and decreased FA values within lesions and normal-appearing brain tissues in SPMS, reflecting widespread demyelination and axonal degeneration. These changes are significantly correlated with neurological disability and cognitive decline, further emphasizing their clinical relevance [38,41].

MRS assesses brain metabolism by quantifying metabolites such as N-acetyl aspartate (NAA, a marker of neuronal integrity), choline (Cho, a marker of membrane turnover and myelin breakdown), creatine (Cr, a marker of gliosis), and myoinositol (mIn, a marker of astroglial activation). Reduced NAA and elevated Cho levels, frequently observed in SPMS, underscore persistent neuronal and myelin damage. These metabolic alterations correlate closely with disease severity, highlighting the potential of this technique as a sensitive biomarker for monitoring SPMS progression [33].

2. Functional MRI.

Functional MRI (fMRI) has emerged as a valuable tool for evaluating connectivity changes in SPMS, providing insights into the functional integrity and adaptability of neural networks as the disease progresses. While conventional MRI is crucial for diagnosing and monitoring structural lesions, fMRI allows for the assessment of functional changes, specifically alterations in resting-state functional connectivity (rsFC), highlighting subtle neural network disruptions that underlie clinical deterioration.

Recent studies employing multiparametric MRI have demonstrated the significance of functional connectivity in understanding cognitive impairment in patients with SPMS. Mistri et al. reported that cognitive dysfunction in SPMS is associated with structural abnormalities and white matter tract involvement, yet interestingly, resting-state functional connectivity did not significantly contribute to global cognitive functioning. This suggests that while structural integrity is critical for cognitive performance, functional connectivity changes may be subtler and require more targeted methods of analysis to detect significant clinical correlations [7].

Other studies suggest a more nuanced relationship between functional MRI changes and clinical outcomes. For instance, Preziosa et al. highlighted that altered rsFC, particularly within frontal cortico-subcortical networks involving the dorsolateral prefrontal cortex, caudate nucleus, and thalamus, correlated with fatigue and dual-task performance impairment, which is common in progressive MS phenotypes. These findings underscore the involvement of frontal networks in mediating the functional deficits seen in SPMS, supporting the hypothesis that specific functional network disruptions underlie the distinct clinical symptoms [17].

Furthermore, Gravesteijn et al. found preliminary evidence of a potential association between higher cardiorespiratory fitness levels and improved functional connectivity within the sensorimotor network, suggesting a protective or adaptive role of physical fitness in maintaining functional brain connectivity in patients with SPMS. This emphasizes the potential therapeutic implications of enhancing physical fitness as a strategy to mitigate functional connectivity disruptions and associated clinical decline in patients with SPMS [42].

3. Brain Atrophy Measurements.

Brain atrophy is a prominent feature of MS, and its patterns and clinical implications differ significantly between SPMS and RRMS patients. Advanced MRI techniques enable the precise quantification of these atrophic changes, offering insights into disease progression and severity.

In SPMS, widespread brain atrophy predominantly affects the cortical gray matter and deep gray matter structures, such as the thalamus, more extensively than in RRMS [6]. Gray matter atrophy in SPMS is strongly correlated with disability progression, cognitive decline, and overall poor clinical outcomes [33]. Cortical atrophy, in particular, is associated with pronounced cognitive impairment and disability, highlighting its utility as a prognostic indicator in patients with SPMS [18].

Comparatively, RRMS exhibits more pronounced inflammatory activity, with lesions frequently showing gadolinium enhancement, while SPMS demonstrates greater neurodegenerative pathology. As RRMS transitions into SPMS, brain volume loss accelerates, becoming more diffuse and involving multiple cortical and subcortical areas, reflecting the neurodegenerative processes underlying SPMS [9,18].

Clinically, measurements of brain atrophy, including whole-brain, cortical, and deep gray matter volumes, have become critical endpoints in therapeutic trials and are recommended for routine clinical monitoring in patients with SPMS. Progressive atrophy predicts disability accumulation and cognitive dysfunction, emphasizing the need for therapies targeting neurodegeneration beyond inflammatory processes alone [34].

4. Emerging Modalities.

Emerging imaging techniques, such as PET-MRI and ultra-high-field MRI (7 Tesla, 7T MRI), have significantly advanced the visualization and understanding of MS pathology, particularly in the challenging diagnosis of SPMS.

PET-MRI integrates functional, structural, and metabolic data, enabling a detailed evaluation of inflammation and neurodegeneration in SPMS. Studies utilizing PET with radioligands like [11C]TMSX and [11C]PK11195 have revealed important insights into molecular alterations in both GM and NAWM. For instance, increased binding of [11C]TMSX to adenosine A2A receptors has been demonstrated in the NAWM of patients with SPMS, correlating strongly with disability [43]. Additionally, PET imaging using [11C]PK11195, which binds specifically to the translocator protein (TSPO) expressed in activated microglia, has allowed the detection of diffuse inflammation in the NAWM and periplaque areas of patients with SPMS, thus revealing chronic inflammatory processes that conventional MRI fails to visualize [44].

Furthermore, PET imaging techniques enable the study of synaptic density and neuronal integrity using advanced tracers such as [11C]UCB-J, highlighting the extent of synaptic loss and dysfunction in patients with SPMS. The utilization of PET-MRI in longitudinal studies promises to elucidate dynamic changes in neuroinflammation and neurodegeneration, contributing significantly to personalized therapeutic strategies and early intervention opportunities [25].

The advent of ultra-high-field MRI at 7 Tesla (7T MRI) has notably improved the visualization of subtle pathological features, including cortical and subcortical GM lesions, which are challenging to detect at lower field strengths. 7T MRI enhances lesion detection sensitivity, especially in cortical areas, and reveals a significantly higher number of cortical lesions than conventional MRI techniques, providing crucial insights into cortical pathology, which is a significant factor in cognitive impairment and disability progression in SPMS [45]. Specifically, 7T MRI can detect cortical lesions with greater spatial resolution, enabling better differentiation of lesion subtypes and detailed characterization of lesion morphology, including the presence of chronic active “smoldering” lesions with inflammatory rims containing iron.

Additionally, 7T MRI facilitates the detection of leptomeningeal inflammation, a pathological feature closely linked to cortical damage and neurodegeneration in SPMS, thus potentially guiding more precise therapeutic targets [25].

Segmentation volumetry through advanced MRI analysis has emerged as a powerful tool for quantifying GM and WM volumes, which is crucial for monitoring SPMS progression. Volumetric segmentation helps identify atrophy patterns, which are particularly extensive in the cortical and deep GM areas during the progressive phase. This technique supports both clinical prognosis and therapeutic monitoring, offering precise and quantitative assessments of the severity of neurodegeneration [25]. Accurate volumetric segmentation can differentiate subtle regional atrophy patterns, such as thalamic and hippocampal volume loss, which are strongly associated with cognitive decline and disability progression.

Moreover, longitudinal volumetric measurements enhance our understanding of the temporal dynamics of neurodegeneration and improve the predictive capability of MRI biomarkers for disease progression. Consequently, volumetric segmentation methods have become integral to clinical trials evaluating the effectiveness of neuroprotective therapies in SPMS.

## 5. Discussion

The findings summarized in this narrative review underscore the central role of MRI in elucidating the key pathological processes associated with SPMS. Compared with RRMS, SPMS involves a greater neurodegenerative burden and fewer overt inflammatory flares, making it particularly challenging to detect early using conventional imaging techniques. The shift toward compartmentalized inflammation and smoldering lesions indicates that MRI measures beyond simple T2-hyperintense lesion volume—such as cortical lesion load, iron rim lesions, and diffuse brain atrophy—carry significant prognostic value.

An important pattern emerging from the literature is that early disability accumulation, especially when coupled with a high lesion burden and progressive brain atrophy, strongly predicts conversion to SPMS. Although cross-sectional studies have established robust correlations between MRI findings and clinical progression, the field still lacks large-scale longitudinal data, specifically tracking advanced MRI markers over time. This gap hampers the formation of definitive predictive models that can reliably identify patients at a high risk of SPMS.

Moreover, the review highlights how advanced techniques, including magnetization transfer imaging, diffusion tensor imaging, and quantitative susceptibility mapping, help characterize microstructural and metabolic changes that are not evident in standard MRI sequences. These enhancements reveal aspects of tissue injury, such as iron deposition in the deep gray matter, which are particularly relevant for SPMS. Nonetheless, these sophisticated tools remain inconsistently adopted, partly because of the lack of standardized protocols across centers and variability in scanner hardware and post-processing methods.

From a clinical standpoint, the specificity of MRI in capturing the structural correlates of decline positions it as an indispensable asset in early intervention strategies. By identifying patients in the early or subclinical phases of progression, clinicians can refine therapeutic decisions, potentially incorporating higher-efficacy disease-modifying therapies or neuroprotective approaches. In this regard, advanced MRI biomarkers could also serve as surrogate endpoints in clinical trials, accelerating the evaluation of novel treatments aimed at mitigating neurodegeneration in patients with ALS.

Future directions should include large-scale prospective collaborations that unify imaging protocols and incorporate advanced MRI markers from the outset. Integration with fluid biomarkers, such as serum neurofilament light chain, could further enrich the predictive power of MRI findings, particularly for distinguishing inflammatory and neurodegenerative contributions to disability. Additionally, a better understanding of how demographic factors (e.g., older onset and comorbid vascular disease) modify MRI signals is paramount for refining the clinical use of these imaging biomarkers.

## 6. Limitations

This review is limited by its narrative design, which carries an inherent risk of selection bias in the literature. Unlike a systematic review or meta-analysis, there was no predefined search protocol, potentially reducing the transparency and reproducibility of our selection of references.

The second key limitation is the heterogeneity of MRI techniques and outcome measures in the available studies. Different research groups employ varying scanner field strengths, sequences (e.g., T2-FLAIR vs. double-inversion recovery), and post-processing methods (e.g., segmentation software for volumetry). This lack of standardization creates challenges in directly comparing results, thus impeding the establishment of universally accepted thresholds for prognostic imaging markers in the future.

Lastly, although advanced MRI modalities like 7T imaging or susceptibility-based sequences show promise, they remain unavailable in many clinical settings, restricting the immediate clinical impact of these findings. The scalability of these techniques, particularly in routine practice, requires further investigation. Consequently, while current evidence underscores the strong potential of MRI to guide SPMS management, translating research insights into everyday care demands broader standardization, larger longitudinal cohorts, and further validation of new imaging biomarkers.

## 7. Conclusions

MRI has proven crucial for identifying and characterizing neurodegenerative changes in SPMS from earlier disease phases. Traditional MRI findings—such as T2 lesion load, T1-hypointense “black holes”, and evidence of spinal cord involvement—correlate strongly with disability progression. Emerging markers, including SELs and iron rim lesions, further underscore the complexity of SPMS pathology by capturing the smoldering inflammation and ongoing tissue damage. However, significant gaps remain, most notably the need for larger longitudinal cohorts and standardized imaging protocols to validate these newer techniques. Broadening the scope of MRI-based biomarkers by integrating measures of gray matter atrophy, microstructural changes, and complementary fluid biomarkers like serum neurofilament light could better elucidate SPMS progression. Such refinements have direct clinical benefits, from guiding therapeutic decisions to improving patient counseling and outcomes. Continued efforts to harmonize advanced imaging methods and incorporate them into routine practice will be key to enhancing the precision of SPMS treatment.

## Figures and Tables

**Table 1:** Key pathophysiological changes in RRMS and SPMS.

Mechanism	RRMS (Inflammation Driven)	SPMS (Neurodegeneration Driven)	Reference
Immune Activation	Peripheral immune cell infiltration (T-cells, B-cells, macrophages)	Chronic compartmentalized inflammation within CNS (microglial activation)	[5]
Lesion Formation	New gadolinium-enhancing lesions due to acute inflammation	Fewer new lesions; smoldering lesions with ongoing damage	[4]
Axonal Damage	Axonal transection in active lesions due to immune attack	Widespread axonal loss due to mitochondrial dysfunction	[16]
Myelin Damage and Repair	Demyelination with some remyelination potential	Extensive demyelination with impaired remyelination	[5]
Cytokine Profile	Increased levels of TNF-α, IFN-γ, IL-17 (pro-inflammatory)	Shift towards IL-6, IL-1β, TNF-α (chronic inflammation)	[4]
Neurodegeneration	Minimal neurodegeneration	Progressive neuronal loss, cortical atrophy	[16]
Mitochondrial Dysfunction	Less prominent	Increased oxidative stress, mitochondrial failure	[4]
Blood-Brain Barrier (BBB)	Frequent BBB disruption allowing immune cell entry	More stable BBB but persistent CNS inflammation	[5]

Abbreviations: RRMS: Relapsing-Remitting Multiple Sclerosis, SPMS: Secondary Progressive Multiple Sclerosis, CNS: Central Nervous System, TNF-α: Tumor Necrosis Factor-alpha, IFN-γ: Interferon-gamma, IL-17: Interleukin-17, IL-6: Interleukin-6, IL-1β: Interleukin-1 beta, BBB: Blood-Brain Barrier.

**Table 2:** MRI Biomarkers in SPMS.

Imaging Biomarker	Type	Pathophysiological Relevance	Clinical Utility	References
**T2-Hyperintense Lesions**	Structural	Demyelination and gliosis in white matter	Total lesion burden correlated with disease duration and relapse history	[2,6]
**T1-Hypointense Lesions (“Black Holes”)**	Structural	Irreversible axonal loss and matrix destruction	Marker of severe neuroaxonal damage; predicts sustained disability	[7,8]
**Brain Atrophy**	Structural	Global and regional neurodegeneration (especially cortical and thalamic atrophy)	Strong predictor of long-term disability and cognitive impairment	[6,9]
**Spinal Cord Lesions/Atrophy**	Structural	Focal demyelination and axonal degeneration in motor tracts	Linked to motor decline and early transition to SPMS	[6,7]
**Slowly Expanding Lesions (SELs)**	Advanced	Chronic active plaques with peripheral expansion and microglial activation	Associated with smoldering inflammation and ongoing tissue destruction	[4,17]
**Iron Rim Lesions**	Advanced	Paramagnetic rims due to iron-laden microglia/macrophages in chronic active lesions	Specific to progressive MS, it correlates with more aggressive disease	[18,32]
**Atrophied Lesion Volume (ALV)**	Quantitative	Represents progressive tissue loss within pre-existing lesions	Stronger correlate with disability than lesion count alone	[7,8]
**DTI (↓FA, ↑MD)**	Microstructural	Loss of white matter tract integrity and axonal disruption	Detects diffuse damage in both lesions and normal-appearing white matter	[7,33]
**Magnetization Transfer Ratio (MTR)**	Microstructural	Decreased interaction between macromolecules and protons indicating demyelination	Sensitive to myelin loss; useful in both lesioned and NAWM regions	[18,34]
**MR Spectroscopy (↓NAA, ↑Choline)**	Metabolic	Reflects reduced neuronal viability and increased membrane turnover	Biochemical evidence of neurodegeneration correlates with clinical severity	[34]

Abbreviations: SELs: Slowly Expanding Lesions, ALV: Atrophied Lesion Volume, DTI: Diffusion Tensor Imaging, FA: Fractional Anisotropy, MD: Mean Diffusivity, MTR: Magnetization Transfer Ratio, NAWM: Normal-Appearing White Matter, NAA: N-Acetylaspartate, SPMS: Secondary Progressive Multiple Sclerosis, MR: Magnetic Resonance, MRI: Magnetic Resonance Imaging, T1: T1-weighted MRI sequence, T2: T2-weighted MRI sequence.

## References

[1] Ciubotaru A., Alexa D., Grosu C., Böckels L., Păvăleanu I., Maștaleru A., Leon M.M., Covali R., Roman E.M., Bistriceanu C.E. (2024). Validation of a Set of Clinical Criteria for the Diagnosis of Secondary Progressive Multiple Sclerosis. Brain Sci..

[2] McKay K.A., Kwan V., Duggan T., Tremlett H. (2015). Risk factors associated with the onset of relapsing-remitting and primary progressive multiple sclerosis: A systematic review. BioMed Res. Int..

[3] Minderhoud J.M., van der Hoeven J.H., Prange A.J. (1988). Course and prognosis of chronic progressive multiple sclerosis. Results of an epidemiological study. Acta Neurol. Scand..

[4] Lassmann H. (2019). Pathogenic Mechanisms Associated with Different Clinical Courses of Multiple Sclerosis. Front. Immunol..

[5] Cree B.A.C., Arnold D.L., Chataway J., Chitnis T., Fox R.J., Pozo Ramajo A., Murphy N., Lassmann H. (2021). Secondary Progressive Multiple Sclerosis: New Insights. Neurology.

[6] Koch M.W., Mostert J., Repovic P., Bowen J.D., Strijbis E., Uitdehaag B., Cutter G. (2022). MRI brain volume loss, lesion burden, and clinical outcome in secondary progressive multiple sclerosis. Mult. Scler. J..

[7] Mistri D., Cacciaguerra L., Valsasina P., Pagani E., Filippi M., Rocca M.A. (2023). Cognitive function in primary and secondary progressive multiple sclerosis: A multiparametric magnetic resonance imaging study. Eur. J. Neurol..

[8] Eshaghi A., Prados F., Brownlee W.J., Altmann D.R., Tur C., Cardoso M.J., De Angelis F., van de Pavert S.H., Cawley N., De Stefano N. (2018). Deep gray matter volume loss drives disability worsening in multiple sclerosis. Ann. Neurol..

[9] Arnold D.L., Piani-Meier D., Bar-Or A., Benedict R.H., Cree B.A., Giovannoni G., Gold R., Vermersch P., Arnould S., Dahlke F. (2022). Effect of siponimod on magnetic resonance imaging measures of neurodegeneration and myelination in secondary progressive multiple sclerosis: Gray matter atrophy and magnetization transfer ratio analyses from the EXPAND phase 3 trial. Mult. Scler..

[10] Confavreux C., Vukusic S., Adeleine P. (2003). Early clinical predictors and progression of irreversible disability in multiple sclerosis: An amnesic process. Brain.

[11] Tedeholm H., Piehl F., Lycke J., Link J., Stawiarz L., Burman J., de Flon P., Fink K., Gunnarsson M., Mellergård J. (2022). Effectiveness of first-generation disease-modifying therapy to prevent conversion to secondary progressive multiple sclerosis. Mult. Scler. Relat. Disord..

[12] Davda N., Tallantyre E., Robertson N.P. (2019). Early MRI predictors of prognosis in multiple sclerosis. J. Neurol..

[13] Sharmin S., Roos I., Simpson-Yap S., Malpas C., Sánchez M.M., Ozakbaş S., Horakova D., Havrdova E.K., Patti F., Alroughani R. (2023). The risk of secondary progressive multiple sclerosis is geographically determined but modifiable. Brain.

[14] Genovese A.V., Hagemeier J., Bergsland N., Jakimovski D., Dwyer M.G., Ramasamy D.P., Lizarraga A.A., Hojnacki D., Kolb C., Weinstock-Guttman B. (2019). Atrophied Brain T2 Lesion Volume at MRI Is Associated with Disability Progression and Conversion to Secondary Progressive Multiple Sclerosis. Radiology.

[15] Comi G., Filippi M., Martinelli V., Campi A., Rodegher M., Alberoni M., Sirabian G., Canal N. (1995). Brain MRI correlates of cognitive impairment in primary and secondary progressive multiple sclerosis. J. Neurol. Sci..

[16] Kuhlmann T., Moccia M., Coetzee T., Cohen J.A., Correale J., Graves J., Marrie R.A., Montalban X., Yong V.W., Thompson A.J. (2023). Multiple sclerosis progression: Time for a new mechanism-driven framework. Lancet Neurol..

[17] Preziosa P., Rocca M.A., Pagani E., Valsasina P., Amato M.P., Brichetto G., Bruschi N., Chataway J., Chiaravalloti N.D., Cutter G. (2023). Structural and functional magnetic resonance imaging correlates of fatigue and dual-task performance in progressive multiple sclerosis. J. Neurol..

[18] Rocca M.A., Preziosa P., Barkhof F., Brownlee W., Calabrese M., De Stefano N., Granziera C., Ropele S., Toosy A.T., Vidal-Jordana À. (2024). Current and future role of MRI in the diagnosis and prognosis of multiple sclerosis. Lancet Reg. Health Eur..

[19] Absinta M., Sati P., Masuzzo F., Nair G., Sethi V., Kolb H., Ohayon J., Wu T., Cortese I.C.M., Reich D.S. (2019). Association of chronic active multiple sclerosis lesions with disability in vivo. JAMA Neurol..

[20] Zohar D.N., Magalashvili D., Dreyer-Alster S., Hoffmann C., Harari G., Dolev M., Achiron A. (2023). Radiological Disease Activity in Secondary Progressive Multiple Sclerosis. Eur. Neurol..

[21] Chisari C.G., Amato M.P., Di Sapio A., Foschi M., Iaffaldano P., Inglese M., Fermo S.L., Lugaresi A., Lus G., Mascoli N. (2024). Active and non-active secondary progressive multiple sclerosis patients exhibit similar disability progression: Results of an Italian MS registry study (ASPERA). J. Neurol..

[22] Inojosa H., Proschmann U., Akgün K., Ziemssen T. (2021). A focus on secondary progressive multiple sclerosis (SPMS): Challenges in diagnosis and definition. J. Neurol..

[23] Lorscheider J., Buzzard K., Jokubaitis V., Spelman T., Havrdova E., Horakova D., Trojano M., Izquierdo G., Girard M., Duquette P. (2016). Defining secondary progressive multiple sclerosis. Brain A J. Neurol..

[24] Scalfari A., Neuhaus A., Daumer M., Muraro P.A., Ebers G.C. (2014). Onset of secondary progressive phase and long-term evolution of multiple sclerosis. J. Neurol. Neurosurg. Psychiatry.

[25] Tavazzi E., Zivadinov R., Dwyer M.G., Jakimovski D., Singhal T., Weinstock-Guttman B., Bergsland N. (2020). MRI biomarkers of disease progression and conversion to secondary-progressive multiple sclerosis. Expert Rev. Neurother..

[26] Masek M., Vaneckova M., Krasensky J., Danes J., Havrdova E., Hrebikova T., Seidl Z. (2008). Secondary-progressive form of multiple sclerosis: MRI changes versus clinical status. Neuroendocrinol. Lett..

[27] Ciubotaru A., Ignat E.B., Alexa D., Grosu C., Păvăleanu I., Manole A., Maștaleru A., Leon M.M., Matei D.V., Azoicăi D. (2024). Influence of Education, Cognition, and Physical Disability on Quality of Life of Romanian Patients with Multiple Sclerosis-A Cohort Study. Medicina.

[28] Zivadinov R., Ramasamy D.P., Vaneckova M., Gandhi S., Chandra A., Hagemeier J., Bergsland N., Polak P., Benedict R.H., Hojnacki D. (2017). Leptomeningeal contrast enhancement is associated with progression of cortical atrophy in multiple sclerosis. Mult. Scler. J..

[29] Bischof A., Papinutto N., Keshavan A., Rajesh A., Kirkish G., Zhang X., Mallott J.M., Asteggiano C., Sacco S., Gundel T.J. (2022). Spinal cord atrophy predicts progressive disease in relapsing multiple sclerosis. Ann. Neurol..

[30] Filippi M., Preziosa P., Barkhof F., Chard D.T., De Stefano N., Fox R.J., Gasperini C., Kappos L., Montalban X., Moraal B. (2021). Diagnosis of Progressive Multiple Sclerosis from the Imaging Perspective: A Review. JAMA Neurol..

[31] Siger M. (2022). Magnetic Resonance Imaging in Primary Progressive Multiple Sclerosis Patients: Review. Clin. Neuroradiol..

[32] Dal-Bianco A., Grabner G., Kronnerwetter C., Weber M., Kornek B., Kasprian G., Berger T., Leutmezer F., Rommer P.S., Trattnig S. (2021). Long-term evolution of multiple sclerosis iron rim lesions in 7 T MRI. Brain A J. Neurol..

[33] Williams T., Tur C., Eshaghi A., Doshi A., Chan D., Binks S., Wellington H., Heslegrave A., Zetterberg H., Chataway J. (2022). Serum neurofilament light and MRI predictors of cognitive decline in patients with secondary progressive multiple sclerosis: Analysis from the MS-STAT randomised controlled trial. Mult. Scler. J..

[34] Weidauer S., Raab P., Hattingen E. (2021). Diagnostic approach in multiple sclerosis with MRI: An update. Clin. Imaging.

[35] Calvi A., Carrasco F.P., Tur C., Chard D.T., Stutters J., De Angelis F., John N., Williams T., Doshi A., Samson R.S. (2022). Association of Slowly Expanding Lesions on MRI with Disability in People with Secondary Progressive Multiple Sclerosis. Neurology.

[36] Magliozzi R., Reynolds R., Calabrese M. (2018). MRI of cortical lesions and its use in studying their role in MS pathogenesis and disease course. Brain Pathol..

[37] Mammi S., Filippi M., Martinelli V., Campi A., Colombo B., Scotti G., Canal N., Comi G. (1996). Correlation between brain MRI lesion volume and disability in patients with multiple sclerosis. Acta Neurol. Scand..

[38] Orbach R., Zhao Z., Wang Y.C., O’Neill G., Cadavid D. (2012). Comparison of disease activity in SPMS and PPMS in the context of multicenter clinical trials. PLoS ONE.

[39] Lomer N.B., Asalemi K.A., Saberi A., Sarlak K. (2024). Predictors of multiple sclerosis progression: A systematic review of conventional magnetic resonance imaging studies. PLoS ONE.

[40] Absinta M., Lassmann H., Trapp B.D. (2020). Mechanisms underlying progression in multiple sclerosis. Curr. Opin. Neurol..

[41] Ananthavarathan P., Sahi N., Chard D.T. (2024). An update on the role of magnetic resonance imaging in predicting and monitoring multiple sclerosis progression. Expert Rev. Neurother..

[42] Gravesteijn A.S., van der Kruit A., Bet M., Beckerman H., Schoonheim M.M., Heuvel O.A.V.D., Vriend C., van Wegen E.E.H., de Jong B.A., de Groot V. (2025). Associations between physical fitness and structural and functional MRI measures in secondary progressive multiple sclerosis: Cross-sectional findings from the exercise PRO-MS study. Mult. Scler. Relat. Disord..

[43] Rissanen E., Virta J.R., Paavilainen T., Tuisku J., Helin S., Luoto P., Parkkola R., Rinne J.O., Airas L. (2013). Adenosine A2A receptors in secondary progressive multiple sclerosis: A [(11)C]TMSX brain PET study. J. Cereb. Blood Flow Metab. Off. J. Int. Soc. Cereb. Blood Flow Metab..

[44] Rissanen E., Tuisku J., Rokka J., Paavilainen T., Parkkola R., Rinne J.O., Airas L. (2014). In Vivo Detection of Diffuse Inflammation in Secondary Progressive Multiple Sclerosis Using PET Imaging and the Radioligand ¹¹C-PK11195. J. Nucl. Med. Off. Publ. Soc. Nucl. Med..

[45] Harrison D.M., Choi S., Bakshi R., Beck E.S., Callen A.M., Chu R., Silva J.D.S., Fetco D., Greenwald M., Kolind S. (2024). Pooled analysis of multiple sclerosis findings on multisite 7 Tesla MRI: Protocol and initial observations. Hum. Brain Mapp..

